# Comparative Analysis of Vascular Mimicry in Head and Neck Squamous Cell Carcinoma: In Vitro and In Vivo Approaches

**DOI:** 10.3390/cancers13194747

**Published:** 2021-09-23

**Authors:** Roosa Hujanen, Rabeia Almahmoudi, Tuula Salo, Abdelhakim Salem

**Affiliations:** 1 Department of Oral and Maxillofacial Diseases, Clinicum, University of Helsinki, 00014 Helsinki, Finland; roosa.hujanen@helsinki.fi (R.H.); rabeia.mustafa@helsinki.fi (R.A.); tuula.salo@helsinki.fi (T.S.); 2 Translational Immunology Research Program (TRIMM), Research Program Unit (RPU), University of Helsinki, 00014 Helsinki, Finland; 3 Helsinki University Hospital (HUS), 00029 Helsinki, Finland; 4 Cancer and Translational Medicine Research Unit, University of Oulu, 90014 Oulu, Finland; 5 Department of Pathology, Helsinki University Hospital (HUS), 00029 Helsinki, Finland

**Keywords:** head and neck squamous cell carcinoma, vascular mimicry, Matrigel, Myogel, zebrafish, metastasis

## Abstract

**Simple Summary:**

Head and neck squamous cell carcinomas (HNSCCs) are common and among the deadliest neoplasms worldwide, wherein metastasis represents the main cause of the poor survival outcomes. Tumour cells require blood vessels in order to grow and invade the surrounding tissues. Recently, a new phenomenon termed vascular mimicry (VM) was introduced, whereby tumour cells can independently form vessel-like structures to promote their growth and metastasis. VM has been characterized in many solid tumours, including HNSCC. A large body of research evidence shows that patients with positive VM exhibit poor treatment response and dismal survival rates. Thus, VM represents a promising therapeutic and prognostic target in cancer. However, there is limited knowledge regarding the identification of VM in HNSCC (in vitro and in vivo) and what factors may influence such a phenomenon. This study aims to address these limitations, which may facilitate the therapeutic exploitation of VM in HNSCC.

**Abstract:**

Tissue vasculature provides the main conduit for metastasis in solid tumours including head and neck squamous cell carcinoma (HNSCC). Vascular mimicry (VM) is an endothelial cell (EC)-independent neovascularization pattern, whereby tumour cells generate a perfusable vessel-like meshwork. Yet, despite its promising clinical utility, there are limited approaches to better identify VM in HNSCC and what factors may influence such a phenomenon in vitro. Therefore, we employed different staining procedures to assess their utility in identifying VM in tumour sections, wherein mosaic vessels may also be adopted to further assess the VM-competent cell phenotype. Using 13 primary and metastatic HNSCC cell lines in addition to murine- and human-derived matrices, we elucidated the impact of the extracellular matrix, tumour cell type, and density on the formation and morphology of cell-derived tubulogenesis in HNSCC. We then delineated the optimal cell numbers needed to obtain a VM meshwork in vitro, which revealed cell-specific variations and yet consistent expression of the EC marker CD31. Finally, we proposed the zebrafish larvae as a simple and cost-effective model to evaluate VM development in vivo. Taken together, our findings offer a valuable resource for designing future studies that may facilitate the therapeutic exploitation of VM in HNSCC and other tumours.

## 1. Introduction

Head and neck squamous cell carcinoma (HNSCC) includes tumours of the oral cavity, hypopharynx, oropharynx, nasopharynx, and larynx [1]. Overall, HNSCC represents one of the most common cancers worldwide with relatively poor survival outcomes that remain stagnant at around 50% [2]. Such dismal prognosis of HNSCC patients has been largely attributed to tumour cell invasiveness and metastasis [3,4]. Thus, a better understanding of the different mechanisms and patterns underlying tumour cell dissemination could improve the management and survival outcomes of HNSCC patients.

Vascular mimicry (VM; a.k.a. vasculogenic mimicry) is a newly described pattern of tumour-related neoangiogenesis, whereby aggressive tumour cells can form tube-like vascular networks independently of endothelial cells [5,6]. These de novo VM structures were first described in patients with aggressive melanoma. Shortly thereafter, myriad studies have revealed many interesting characteristics of VM in various cancers including HNSCC [7,8]. In addition to satisfying the nutrient need of the primary tumour, VM is believed to provide tumour cells with an alternative route to intravasate and undergo metastasis [9,10]. In this regard, VM was shown to efficiently drive tumour cell metastasis in a polyclonal mouse model of breast cancer [10]. Furthermore, several studies revealed significant association between VM and lymph node metastasis (LNM) and hence worse prognosis in numerous malignancies [11,12,13]. We showed in a recent meta-analysis study that HNSCC patients with VM^+ve^ tumours had shorter overall survival and worse clinicopathological features, including LNM, compared with the VM^-ve^ group [8].

A vessel-like structure expressing CD31^-ve^/periodic acid–Schiff (PAS)^+ve^ staining is often considered the “golden” standard to identify VM in histological samples [14]. However, in spite of the spirited debate ignited by this phenomenon, characterizing VM in patient samples has recently drawn criticism for its limitations. On the one hand, such CD31^-ve^/PAS^+ve^ structures may represent irrelevant glycogen-rich areas rather than true mimetic vessels [14,15,16]. On the other hand, the mosaic vessels, concurrently expressing endothelial and tumour cell markers, have been overlooked in HNSCC-related studies, which show limited approaches to identify VM both in vitro and in vivo [7,8]. Therefore, we conducted a comprehensive comparative analysis of VM formation in HNSCC using a variety of procedures. We also proposed the zebrafish larvae as a feasible tool to model VM formation in vivo.

## 2. Materials and Methods

### 2.1. Patient Samples

This study was approved by the National Supervisory Authority for Welfare and Health (VALVIRA) and the Ethics Committee of the Northern Ostrobothnia Hospital District. Our study comprised patients diagnosed with oral tongue SCC (OTSCC) who had undergone surgery in Oulu University Hospital during the period 1990–2010. Formalin-fixed paraffin-embedded (FFPE) samples (*n* = 30) were obtained from the pathology department of Oulu University Hospital. None of these patients had received other prior treatments.

### 2.2. CD31 and PAS Double Staining

The FFPE specimens were deparaffinized and rehydrated and subjected to heat-induced antigen retrieval using Micromed T/T Mega Microwave Processing Lab Station (Hacker Instruments & Industries). Non-specific binding was blocked with Dako peroxidase blocking solution S2023 for 15 min (Dako, Glostrup, Denmark), followed by incubation in a 1:100 polyclonal rabbit anti-CD31 antibody (ab28364; Abcam, Cambridge, UK) for 1 h. Sections were then incubated with horseradish peroxidase for 30 min; treated with DAB (Pierce™ DAB Substrate Kit; Thermo Fisher Scientific; Waltham, MA, USA) for 5 min; and incubated with 0.5% freshly made periodic acid for 10 min. Sections were further stained with Schiff solution for 15 min and rinsed under running water for another 15 min. Slides were incubated with Cole’s hematoxylin for 6 min and mounted in Mountex (HistoLab, Gothenburg, Sweden). All incubations were conducted at room temperature.

### 2.3. Double-Labelling Immunofluorescence (IF)

Following deparaffinization and heat-induced antigen retrieval, sections were blocked for 1 h with 10% donkey normal serum (Sigma-Aldrich; St. Louis, MO, USA). Sections were then incubated overnight with a primary antibody solution containing 1:50 polyclonal rabbit anti-CD31 antibody (ab28364, Abcam, Cambridge, UK) and 1:100 monoclonal mouse antihuman pan-cytokeratin (CK) (M3515, Dako, Glostrup, Denmark) at 4 °C. The following day, the sections were incubated in (1) 1:200 donkey anti-mouse Alexa Fluor^®^-568 or donkey anti-rabbit Alexa Fluor^®^-488 conjugated secondary antibodies (Vector Laboratories; Burlingame, CA, USA) for 1 h and (2) 4′,6-diamidino-2-phenylindole (DAPI; 1:1000; Sigma-Aldrich, St. Louis, MO, USA) for 10 min, and mounted with ProLong^®^ Gold Antifade Mountant (Thermo Fisher Scientific; Waltham, MA, USA). To stain the cell-derived tubular networks, matrix-coated coverslips were fixed for 20 min in 4% paraformaldehyde (PFA; Santa Cruz Biotech., Santa Cruz, CA, USA) and then staining was continued as above. All steps were performed at room temperature unless otherwise indicated. For multiplexed immunohistochemistry (mIHC), the following antibodies were used: 1:50 polyclonal rabbit anti-CD31 antibody (ab28364, Abcam, Cambridge, UK); 1:100 monoclonal mouse anti-CD44 antibody (144M-95; Cell Marque, Rocklin, CA, USA); 1:100 monoclonal mouse antihuman E-cadherin antibody (M361201, Dako, Glostrup, Denmark); monoclonal mouse anti-CK c11 (ab7753, Abcam, Cambridge, UK); and 1:150 monoclonal mouse anti-CK (AE1/AE3; Dako, Glostrup, Denmark). mIHC was performed in the Digital Microscopy and Molecular Pathology Unit (FIMM Institute, University of Helsinki) as described previously [17].

### 2.4. Cell Line and Culture

Thirteen primary and metastatic HNSCC cell lines were used, including HSC-3 (JCRB 0623; Osaka National Institute of Health Sciences, Japan), SCC-25 (ATCC, Rockville, MD, USA) and SAS (JCRB-0260). Ten cell lines (UT-SCC, hereafter SCC) were established directly from the patient biopsy material at the Department of Otorhinolaryngology, Head and Neck Surgery Unit, Turku University Hospital (Table S1). Of these, paired primary and metastatic cell lines (SCC-24A and -24B, respectively) were obtained from the same patient. The SCC-28 cell line was derived from a primary tumour that was first treated with radiotherapy prior to surgical resection. Cancer cell lines were cultured in 1:1 DMEM-F12 medium (Gibco/Invitrogen, Tokyo, Japan) supplemented with 10% heat-inactivated fetal bovine serum (Gibco), penicillin–streptomycin (15140-122, Thermo Fisher Scientific; Waltham, MA, USA), 50 µg/mL ascorbic acid (A1052, AppliChem, Chicago, IL, USA), 250 ng/mL amphotericin B (A2942, Sigma-Aldrich, St. Louis, MO, USA) and 0.4 µg/mL hydrocortisone (H0888, Sigma-Aldrich, St. Louis, MO, USA). Cell lines were maintained in a 95% humidified incubator of 5% CO_2_ at 37 °C. Human umbilical vein endothelial cells (HUVEC; Thermo Fisher Scientific; Waltham, MA, USA) were used as a positive control for the in vitro tubulogenesis. HUVECs were cultured in 200PRF medium supplemented with a low serum growth supplement (Thermo Fisher Scientific; Waltham, MA, USA).

### 2.5. Murine and Human-Derived 3D Matrices

We used the commercial mouse Engelbreth–Holm–Swarm (EHS) sarcoma matrix, Matrigel (Corning, NYC, NY, USA). In addition, we used our in-house gelatinous soluble matrix “Myogel” that is derived from human leiomyoma tissue [18,19]. The preparation and usage of human leiomyoma tissue have been approved by the Ethics Committee of Oulu University Hospital (no. 35/2014). Liquid handling was performed using MultiFlo^™^ FX automated multi-mode reagent dispenser (BioTeK, Winooski, VT, USA).

### 2.6. In Vitro Tube Formation Assay

The in vitro tube formation assay was performed according to a previously published protocol [20]. For the Matrigel-based assay, following a slow overnight thawing at 4 °C, 50 µL of Matrigel was dispensed into a 96-well plate and incubated for 30 min at 37 °C. Cancer cells and HUVECs were detached from 75 cm^2^ flasks (Sigma-Aldrich, St. Louis, MO, USA) with trypsin–EDTA, resuspended in serum-free DMEM or 200PRF medium, and then counted using Scepter^™^ 2.0 Cell Counter (Merck Millipore, Burlington, MA, USA). Cells were seeded on the top of Matrigel at a starting density of 20 × 10^3^ in 50 µL serum-free medium and incubated at 37 °C.

For the Myogel-based assay, the optimal gel concentration (1 mg/mL) was determined using pilot experiments with HUVECs. The Myogel-fibrin matrix was prepared with serum-free medium using the following concentrations: 1 mg/mL Myogel, 1 mg/mL fibrinogen (341578, Merck, Darmstadt, Germany), 66.67 µg/mL aprotinin (A6279-10ML, Merck, Darmstadt, Germany), and 0.6 U/mL thrombin (T6884-100UN, Sigma-Aldrich, St. Louis, MO, USA). To test the potential effects of different gel constituents, Myogel was also combined with 1 mg/mL rat-tail type I collagen (354236; Corning, NYC, NY, USA) or 1–2% low-melting agarose (LMA; 50101, Lonza, Basel, Switzerland). In total, 50 µL of LMA was slowly pipetted into a 96-well plate to avoid bubbles and incubated overnight at 37 °C. The next day, Myogel was pipetted with the cells into the LMA-coated wells. The matrix-coated well plates were incubated for 12 and 24 h for endothelial- and tumour cell-derived tubulogenesis, respectively. The wells were then rinsed in phosphate-buffered solution (PBS), fixed for 20 min with 4% PFA, and stored in 4 °C.

### 2.7. Zebrafish Larvae Assays

In vivo zebrafish experiments were performed in the zebrafish core facility at the University of Helsinki. All procedures were approved by the ethical committee of the regional state administrative agency (license ESAVI/13139/04.10.05/2017). Two-day post-fertilization zebrafish larvae were dechorionated and anaesthetized using 0.04% Tricaine (*n* = 10 per matrix group). Fluorescently labelled with CellTrace^™^ Far Red (Thermo Fisher Scientific; Waltham, MA, USA), HSC-3 cells were washed in PBS and resuspended in 1:1 Matrigel or Myogel, and then microinjected into the perivitelline space using glass microinjection needles (about 1000 cells). Fish were maintained at 34 °C within an embryonic medium (Sigma-Aldrich, St. Louis, MO, USA) for 72 h and then collected, fixed with 10% PFA, and mounted using SlowFade Gold Antifade (Invitrogen, Carlsbad, CA, USA).

### 2.8. Imaging and Tube Formation Analysis

For experiments on tube formation, samples were photographed with magnifications of 4×, 10× and 20× using the reverse Nikon Digital Sight DS-U3 microscope (Nikon, Tokyo, Japan). Each experiment was repeated at least three times independently. Stained section images were acquired with magnifications of 10×, 20×, and 40× using a Leica DM6000 microscope (Leica Microsystems, Wetzlar, Germany). Imaging of zebrafish larvae was performed at the Biomedicum Imaging Unit (University of Helsinki) using a Leica TCS SP8 confocal microscope. The ImageJ software (Wayne Rasband, National Institute of Health, Bethesda, MD, USA) was used by applying the “Angiogenesis Analyzer” plugin to measure several different parameters for evaluating the tube formation as described in the Results section.

## 3. Results

### 3.1. Utility of the CD31^-ve^/PAS^+ve^ Reaction in Identifying the VM in HNSCC Patients

**Figure 1:**
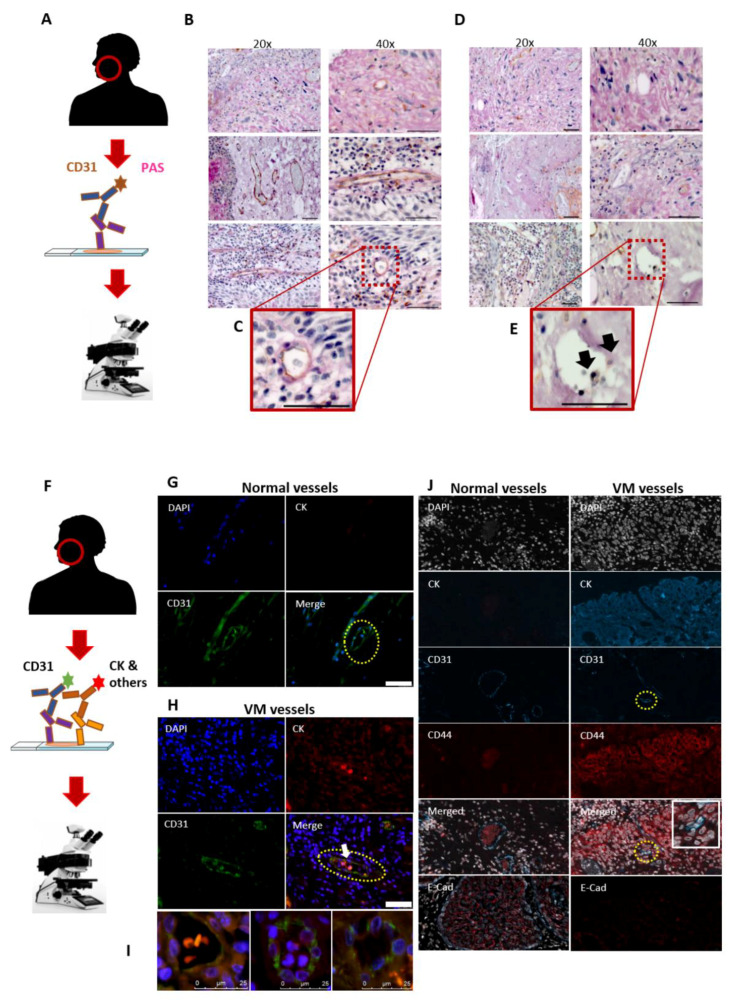
Identification of vascular mimicry (VM) in tumour tissues. (**A**) Representative figures from tumour sections (*n* = 30) obtained from patients with oral tongue squamous cell carcinoma (OTSCC) and stained using a combination of endothelial cell (EC) marker (CD31) and periodic acid–Schiff (PAS) staining. (**B,C**) Normal blood vessels express CD31^+ve^/PAS^+ve^ (brown/pink). Scale bar: 50 µm (**D,E**) Additionally, some CD31^-ve^/PAS^+ve^ vessel-like structures (pink) were identified. However, identifying these structures was often demanding due to the presence of necrotic areas or a faint CD31 signal (black arrows). Scale bar: 50 µm. (**F**) The double-labelled immunofluorescence assay was employed using a combination of CD31 and tumour cell marker (pan-cytokeratin, CK) to investigate the presence of mosaic vessels in HNSCC sections. (**G**) EC-lined blood vessels were easily distinguished in the peritumoral areas (dashed yellow line). Scale bar: 50 µm (**H**) The intratumoral CD31^+ve^/CK^+ve^ mosaic vessels were also observed (dashed yellow line; white arrow). Scale bar: 50 µm. (**I**) These mosaic lumens were either containing RBCs, metastasizing tumour cells or clear. Scale bar: 25 µm. (**J**) The multiplexed immunohistochemistry (mIHC) platform was used to identify VM (merged; inset) as well as to explore the phenotype of VM-forming tumour cells. A negative or weak staining of CD44 was observed in morphologically normal tissues (left), while a strong staining was detected in the VM-forming regions (right). By contrast, E-cadherin (E-Cad) staining was evident in normal and VM-free cancerous tissues (left, red), while it was faint around the mosaic vessels (right). The mIHC images were taken at a magnification of 63×.

To identify the VM in the patient samples, we first employed the traditional staining method—a combination of the endothelial cell marker and PAS staining on FFPE sections from HNSCC patients (Figure 1A). PAS stains basement membrane components such as laminin, collagen, and glycogen, whereas CD31 was opted as a specific endothelial cell marker. Areas of PAS^+ve^ laminin and collagen-rich networks (pink) with the surrounding tumour cells were recognized in the patient samples. Additionally, CD31^+ve^/PAS^+ve^ endothelial vessels (brown/pink; Figure 1B,C) and CD31^-ve^/PAS^+ve^ areas (pink; Figure 1D) were identified. However, it was often onerous to accurately identify the CD31^-ve^/PAS^+ve^ structures due to the presence of necrotic areas or faint CD31 signals that can be easily masked by the surrounding PAS staining (Figure 1E; arrows show a faint signal of CD31).

### 3.2. The Mosaic VM Pattern Reveals Tumour Cell Plasticity

Recently, we showed that oral tongue squamous cell carcinoma (OTSCC) cells express considerable levels of the endothelial marker CD31 in vitro [21]. Due to the limited utility of the CD31^-ve^/PAS^+ve^ reaction in identifying VM in HNSCC tissues, we sought to explore the presence of intratumoral mosaic vessels using CD31^+ve^/CK^+ve^ double-labelled immunofluorescence (Figure 1F). Normal blood vessels were easily distinguished as CD31-expressing lumens mainly in the peritumoral stroma (Figure 1G). Interestingly, OTSCC patient samples revealed distinct and clear intratumoral CD31^+ve^/CK^+ve^ mosaic VM lumens, which also contain red blood cells (Figure 1H, arrow). It has been well reported that VM formation is associated with phenotype switching or “cell stemness” (i.e., tumour cell plasticity), which is mediated by certain events such as upregulation of CD44 and loss of epithelial cell markers including E-cadherin [22,23]. This observation prompted us to explore whether the mosaic vessels can also be harnessed to examine the status of these phenotype mediators. Importantly, using the mIHC platform, the mosaic CD31^+ve^/CK^+ve^ structures revealed an induced CD44-immunoreactivity, while E-cadherin staining was noticeably weaker around the mosaic vessels compared with tumoral VM-free regions (Figure 1I).

### 3.3. Metastatic HNSCC Cells Preferentially form VM in Matrigel

**Figure 2:**
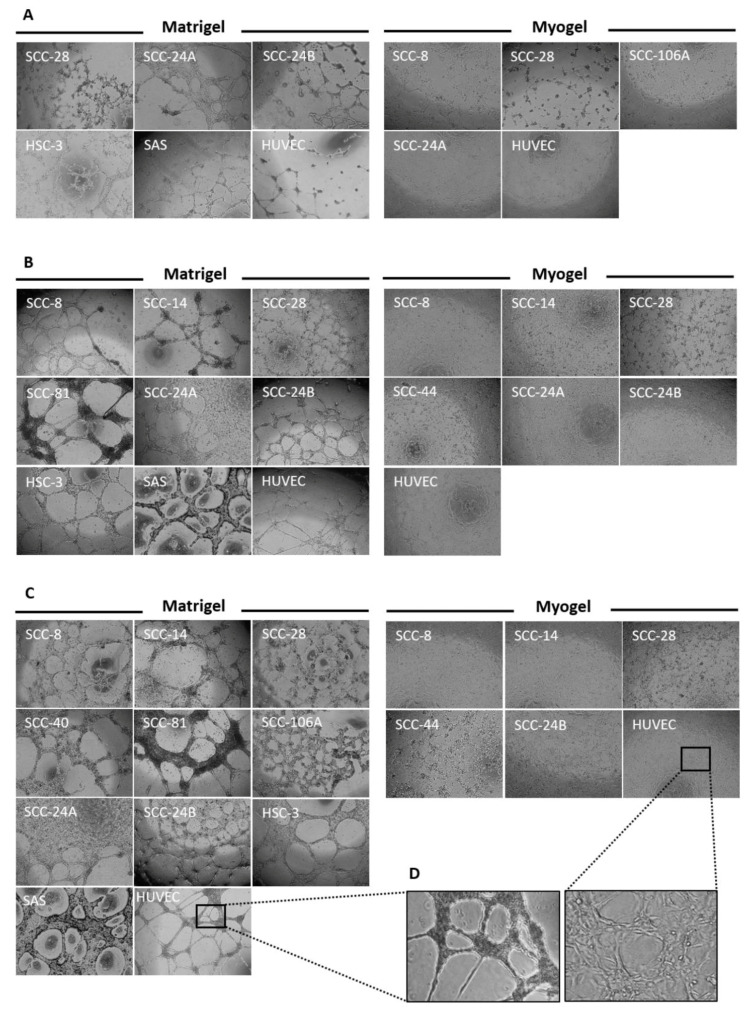
Assessment of tumour cell-derived tubulogenesis in vitro. (**A**) Thirteen cell lines derived from head and neck squamous cell carcinoma (HNSCC) and normal human endothelial cells (HUVEC) were seeded at a starting cell density of 20 × 10^3^ cells on Matrigel or Myogel. All of the highly metastatic HNSCC cell lines (namely, SCC-24B, HSC-3, and SAS) formed a VM meshwork in Matrigel only, while more primary cell lines formed such tubes in Myogel (*n* = 4). HUVEC formed tubes in both matrices. (**B**) At a higher starting cell density of 40 × 10^3^, all metastatic and some primary HNSCC cell lines (*n* = 7) developed longer and more interlaced VM meshwork in Matrigel. In Myogel, primary tumour cell lines (*n* = 5) continued to form VM structures with the most extensive meshwork attained by cells originating from the floor of the mouth and gingiva (SCC-28 and SCC-44, respectively). Additionally, the metastatic cell line (SCC-24B) started to initiate consistent tubes in Myogel that were more extensive than its primary counterpart (SCC-24A). (**C**) When the starting cell density reached 60 × 10^3^, only metastatic and merely two primary cell lines continued to develop thicker and longer VM networks in Matrigel compared with four primary cell lines in Myogel. (**D**) The HUVEC meshwork has become shorter and more interlaced in Myogel. The images were taken at a magnification of 4×.

Previous pioneering studies have shown that cancer cells can form VM capillary networks similar to the endothelial tubulogenesis when cultured on a collagen-rich matrix [24]. However, there is very limited knowledge concerning the effect of the extracellular matrix (ECM) on such a phenomenon. Therefore, after identifying the VM structures in patient samples, we explored whether matrix origin and constituents can influence VM formation in vitro. To this end, 13 primary and metastatic HNSCC cell lines plus HUVEC were seeded on murine- and human-derived matrices at a density of 20 × 10^3^ cells/well, as described previously [20]. Of note, all cell lines with high metastatic potential (*n* = 3) formed capillary networks in Matrigel but not in Myogel. By contrast, the primary cell lines showed a greater tendency to form VM in Myogel (*n* = 4) compared with Matrigel (*n* = 2; Figure 2A). Nevertheless, HUVEC formed consistent tubes in both matrices, suggesting that ECM could be an important modulator of the tumour cell-derived tubulogenesis. At this cell density, the tubes were, however, poorly networked and occupied less than half of the matrix area and were then scored (+) as illustrated in Table 1. Combining Myogel with collagen I or LMA did not noticeably alter VM formation.

### 3.4. Tumour Cell Density Influences VM Formation In Vitro

Tumour cell-derived tubulogenesis is an important assay not only for assessing VM formation but also for testing potential anti-angiogenic drugs in vitro [21]. It is therefore important to discern the optimal number of tumour cells needed to establish mature capillaries for HNSCC-related studies. For this purpose, HNSCC cell lines were seeded on Matrigel or Myogel using different starting cell densities of 20, 40, and 60 × 10^3^ cells. Notably, all metastatic and some primary HNSCC cell lines (*n* = 7) formed longer and well-networked VM structures at 40 × 10^3^ cells, which covered approximately half of the Matrigel (score ++; Figure 2B). Furthermore, only metastatic (*n* = 3) and merely two primary cell lines developed thicker and longer capillary networks when tumour cell density reached 60 × 10^3^, which spread to more than half of the Matrigel (score +++; Figure 2C). In Myogel, primary tumour cell lines (*n* = 5) continued to form VM structures with the most extensive networks attained by cells originating from the floor of the mouth and gingiva (SCC-28, grade 1; SCC-44, grade 3, respectively; Figure 2B,C). Of interest, at higher cell densities, the metastatic cell line (SCC-24B) started to initiate consistent VM networks in Myogel that were more extensive than its primary counterpart (SCC-24A; Figure 2B,C). Moreover, the HUVEC meshwork has apparently become shorter and more interlaced in Myogel (Figure 2D). Two primary cell lines (SCC-73 and SCC-25) failed to form VM networks in either matrices, which remained dispersed in the matrices as round cell aggregates (Figure S1). The scores of VM formation using different cell densities and matrices are listed in Table 1.

**Figure 3:**
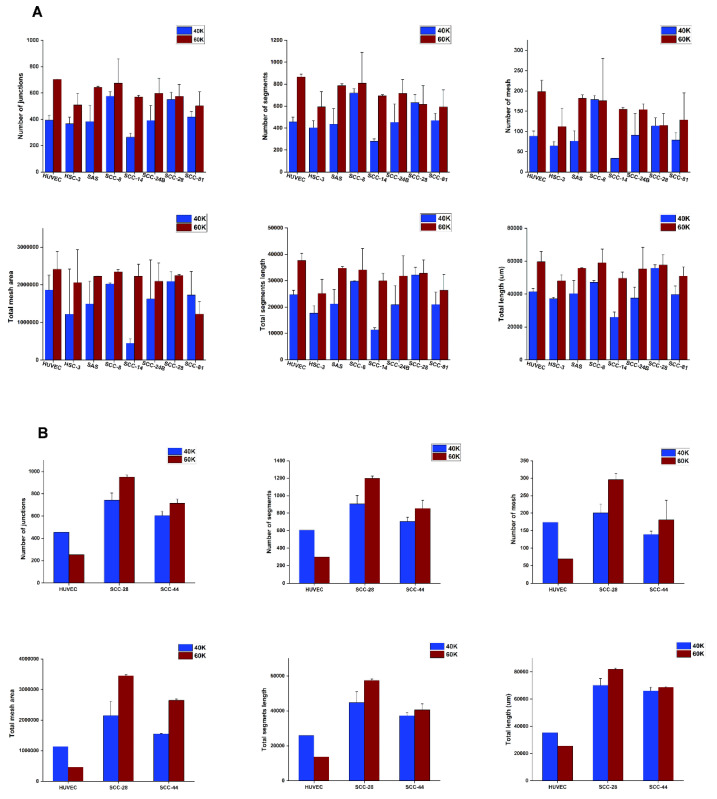
In vitro tube formation analysis of tumour cell-derived vascular mimicry (VM). (**A**) The Angiogenesis Analyser plugin was used to discern the optimal starting cell density needed to establish VM meshwork in vitro. Different tube formation parameters were analysed, including the number of junctions, segments, meshes, total mesh area, total segment length, and total branching lengths of the tubular networks. A starting cell density of 60 × 10^3^ produced consistent mature looping patterns in Matrigel for almost all the included cell lines. However, overall, the analysis shows that both 40 and 60 × 10^3^ concentrations are sufficient to initiate VM structures in vitro. (**B**) The results were comparable in Myogel, with better tubes formed with a starting cell density of 60 × 10^3^. However, at such a higher density, HUVEC meshwork became more extensively interlaced in Myogel, which limited the analyser’s capacity to recognize smaller tubular areas as shown in the figure. Data from one representative experiment, presented as mean ± SD of three technical replicates, are shown.

Next, we used the Angiogenesis Analyser tool to assess the comparative capacity of various HNSCC cells in forming VM capillaries in vitro [25]. Angiogenic parameters including the number of junctions (branching capillary nodes), segments (capillaries delimited by two junctions), meshes (areas enclosed by segments), total meshes area (sum of mesh areas), total segments length (length sum of all segments), and total branching length were quantified. It is worth noting that the pre-irradiated SCC-28 cells formed unique “spiky” capillaries that spread evenly on Matrigel, regardless of their cell density (Figure 3A). Overall, starting cell densities of 40 and 60 × 10^3^ cells were both adequate for initiating proper tubulogenesis in vitro; however, the latter density produced more looping meshwork in most cell lines (Figure 3A,B).

### 3.5. In Vitro VM Networks Reveal Different Morphological Patterns

**Figure 4:**
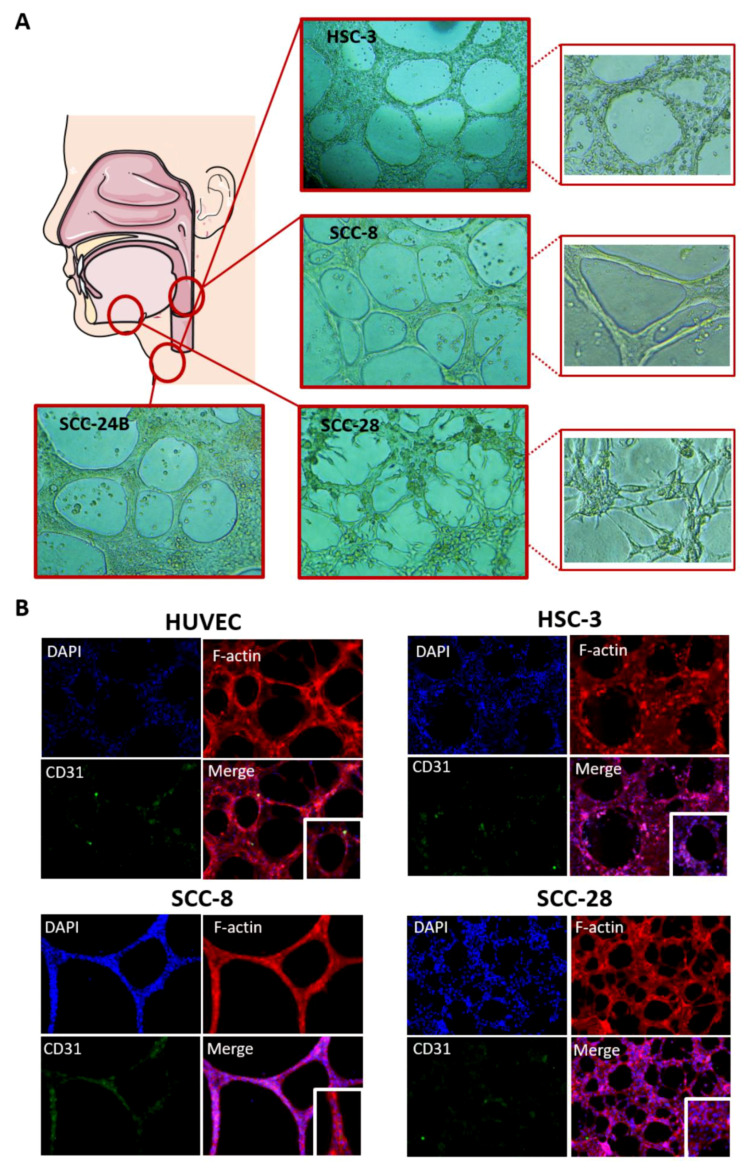
(**A**) Tumour cells show distinct morphological patterns of tubulogenesis in vitro. The highly metastatic tongue cancer cell lines (HSC-3 and SCC-24B) formed the classical “honeycomb-like” looping pattern, while the larynx-derived cell line (SCC-8) attained thinner branches with smoother capillaries. Evidently, cells from the floor of the mouth (SCC-28) formed peculiar and “spike-like” networks, on both Matrigel and Myogel, that were morphologically different from any other cell line. (**B**) In vitro tumour cell-derived tubulogenesis showed substantial resemblance to the endothelial ones. These cell networks expressed the endothelial cell marker CD31, which was primarily localized in the capillary extensions and junctions. The images were taken at magnifications of 10× and 20×.

Interestingly, tumour cells from different head and neck regions formed varying morphological patterns of VM networks in their respective matrix. While metastatic OTSCC cell lines (e.g., HSC-3 and SCC-24B) had the typical “honeycomb-like” pattern, the larynx-derived primary cell line (SCC-8) attained thinner and somewhat smoother capillary extensions (Figure 4A). On the other hand, cells derived from the floor of the mouth (i.e., SCC-28) formed peculiar capillary networks with thick “spike-like” projections in the two matrices (Figure 4A).

### 3.6. In Vitro VM Networks Express Endothelial Cell Marker

Having determined the optimal cell density to establish VM in 3D matrices, we next sought further in vitro verification that HNSCC cells, rather than endothelial cells, were responsible for the observed mosaic pattern in the clinical samples. Hence, tumour cell-derived VM networks on phenol red-free Matrigel were stained with the endothelial cell marker CD31. To unambiguously localize CD31 in relation to tumour cell junctions, VM capillaries were also labelled with Phalloidin–Alexa-594 to stain F-actin networks. Interestingly, tumour cell-derived VM capillaries clearly expressed CD31, which was mostly localized in the tubular extensions and around the capillary junctions (Figure 4B).

### 3.7. Larval Zebrafish as a Novel In Vivo Model for VM Formation

**Figure 5:**
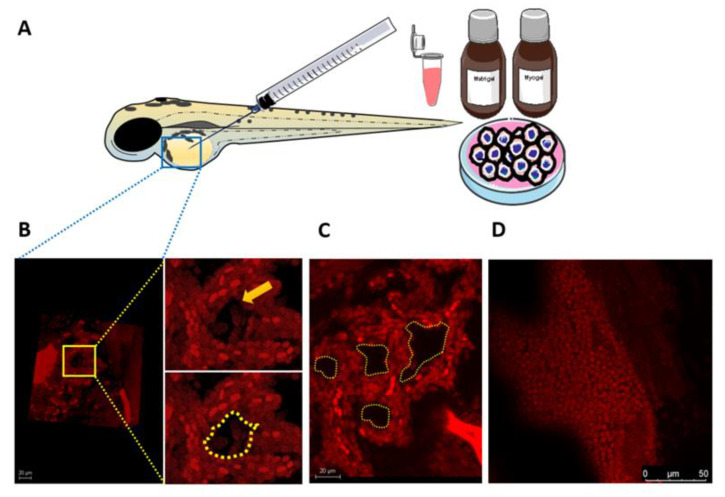
Larval zebrafish model to evaluate vascular mimicry (VM) formation in vitro. (**A**) Fluorescently labelled highly metastatic tumour cells (HSC-3) were resuspended in Matrigel or Myogel and microinjected into the perivitelline space of 2-day-old zebrafish larvae. Fish were screened 27 h post-injection using confocal microscopy. (**B**,**C**) HSC-3 cells formed seemingly VM-like structures in the Matrigel-injected fish. (**D**) No similar tube formation was detected in Myogel-containing fish, supporting a similar outcome from the in vitro assays. Scale bars: 20 and 50 µm.

Testing VM formation in vivo is currently conducted in patient-derived murine xenografts [24]. However, such models can present substantial challenges, including time consumption and cost and labour intensiveness. Therefore, we assessed the utility of zebrafish larvae as a simple and yet efficient approach to optically screening the formation of VM structures in vivo. Fluorescently labelled aggressive tumour cells (HSC-3) were resuspended in their respective matrix and microinjected into the perivitelline space of anaesthetized zebrafish (Figure 5A). Using confocal microscopy at 72 h post-injection, the xenografted tumour cells displayed VM-like structures in some of the fish (*n* = 3) belonging to the Matrigel-injected group, which attained singular or multi-tubular pattern (Figure 5B,C). By contrast, no similar structures were observed in the Myogel-injected group (Figure 5D).

## 4. Discussion

The VM has been well documented in a variety of cancers and is associated with a stem-like cell phenotype, aggressive disease course, and dismal survival outcomes [9,10,13,26]. However, the currently available approaches to identify VM in HNSCC are rather limited, thereby necessitating more research on this intriguing phenomenon [7,8]. In this study, we first revealed some challenges associated with identifying VM in HNSCC sections, wherein the mosaic vessels could be adopted to further assess the phenotype of VM-forming cells. Next, we reported the impact of ECM origin, tumour cell type, and density on the formation and morphology of HNSCC cell-derived tubulogenesis. We then delineated the optimal cell numbers needed to obtain such tubular meshwork in vitro, which also expressed the specific endothelial cell marker—CD31. Finally, we proposed for the first time a simple animal model, the zebrafish larvae, for assessing the development of VM in vivo.

Histologically, VM structures are often identified in cancer patients as PAS^+ve^, RBC-containing, lumen-like structures combined with a negative staining of an endothelial cell marker [14]. However, PAS stains various ECM components including collagens, laminin, and proteoglycans and hence may not always represent the vascular mimetic structures. Using an X-ray microtomography 3D reconstruction, Racordon et al. showed that many PAS^+ve^ areas do not display actual lumens in vitro and may instead represent glycoprotein-rich regions [16]. It has therefore been recommended to be attentive when scoring PAS^+ve^ areas to differentiate VM from non-specific ECM aggregates [7,14]. Furthermore, a strong PAS staining may conceal the expression of endothelial cell markers, making it challenging to discern CD31^-ve^/ PAS^+ve^ patterns. In a different approach, several reports described the existence of “mosaic” vessels expressing both tumour and endothelial cell markers in cancer tissues, emphasizing the importance of tumour cell plasticity in VM formation [6,27,28,29]. Initially, these vessels were thought to result from endothelial and tumour cell merging in blood vessel walls. However, it was later shown that tumour cells are able to form and maintain blood vessels by expressing neuropilin-2, EphA2, and laminin-15γ2 [28]. An interesting study revealed that 20–90% of the vascular endothelium in glioblastoma was derived from VM-forming tumour cells in mice; their selective targeting resulted in tumour reduction and degeneration [26]. Supporting these findings, Kim et al. found that the intratumoral VM channels were derived from CD31^+ve^/CD34^+ve^ gastric tumour cells [30]. Furthermore, we recently showed, by fluorescence-activated cell sorting, that 90% of the HSC-3 cells were CD31^+ve^, compared with 96% of HUVEC [21]. In this study, we manifested this expression phenotypically by showing that tumour cell-derived tubes are CD31^+ve^ with a striking resemblance to the endothelial ones. These findings suggest that intratumoral mosaic vessels may represent an additional staining approach to identifying patterned VM structures in cancer tissues.

Using the mIHC platform, the adhesion molecule CD44—a transmembrane glycoprotein receptor known to promote tumour cell plasticity—and VM were induced around the mosaic VM-forming cells [31]. A tumour cell plasticity is best seen in crucial metastatic processes such as epithelial mesenchymal transition, wherein tumour cells lose their adhesion, polarity, and epithelial cell markers including E-cadherin [32]. It is therefore interesting that mosaic VM-forming regions revealed a faint expression of E-cadherin compared with other epithelial regions. Our results advocate the use of mIHC for the simultaneous assessment of different markers associated with the development of VM.

Previous seminal works on VM have shown that aggressive cancer cells can form tubular networks when seeded on Matrigel. However, it is worth noting that considerable variations exist among different matrices based on their origin, composition, and consistency. Our findings suggest that tumour cell-derived tubulogenesis could be influenced, in part, by the matrix type. Although it is not yet clear why tumour cells have a matrix-specific ability to form VM, variations in ECM features may underpin this interesting observation. For instance, the protein composition of Myogel is substantially different from other EHS-based matrices. Further, crucial carcinogenesis-related properties, such as tumour cell invasion and response to HNSCC-targeted therapy, were more efficiently represented in Myogel than in Matrigel [18,33]. A fascinating observation is that a primary cell line (SCC-24A) formed merely a few tubes compared with an extensively interlaced network formed by its metastatic counterpart (SCC-24B), albeit both were established from the same patient. This confirms previous studies showing that VM is associated with metastatic and highly aggressive tumours. Additionally, we infer that VM competence can differ even within the same patient, signifying the need for more precise targeting of anti-angiogenic therapies. The spike-like pattern formed by tumour cells from the floor of the mouth is another intriguing observation. Interestingly, this particular cell line (SCC-28) was established from a tumour that was treated with radiotherapy prior to surgical resection. In this context, there is abundant evidence that ionizing radiation targeting cancer cells may enhance their metastatic process [34]. Additionally, the floor of the mouth is the most high-risk site for metastasis in oral cancer patients. Thus, it has been recommended that patients with SCC in this site should be offered an elective neck dissection even at early stages of the disease [35]. We encourage further research to investigate whether this peculiar tubular morphology plays a role in the metastatic potential of HNSCC and whether radiation therapy could impact VM formation. Consistent with previous data, endothelial cells formed shorter and much interlaced networks in Myogel [18].

Cancer cell-derived tubulogenesis is a valuable assay not only to evaluate VM formation in vitro but also for testing potential anti-angiogenic therapeutic approaches in HNSCC [21]. Therefore, we presented an easy standardized protocol to establish mature capillary networks using a good number and a variety of HNSCC cell lines. Previous studies reported similar approaches to estimating such an optimal tumour cell number, for instance, in human ovarian cancer cell lines [16]. In this study, authors provided solid evidence that VM tubes in vitro represent in most cases functional hollow channels. Additionally, 15 and 75 × 10^3^ starting numbers of the ovarian cancer cells produced clear tubular formation on day 4 of the experiment. In another protocol, Francescone et al. suggested a starting density of 10 − 20 × 10^3^ cells using melanoma, glioblastoma, and breast cancer cell lines [20]. In the present HNSCC cell lines, comparable starting cell densities (20, 40, and 60 × 10^3^ cells) were used to obtain VM channels within 24 h in culture, confirming that this phenomenon could vary based on the tumour cell type.

Zebrafish larvae have recently emerged as a popular in vivo model of HNSCC to mimic key tumorigenic events such as metastasis [36]. Indeed, zebrafish provides many advantages over other animal models considering its efficiency, feasibility, and cost- and labour-effectiveness [37]. Currently, most in vivo model systems of VM are conducted in murine xenografts. However, in addition to cost and labour challenges, screening of VM in these models can be made only post-mortem, restricting further follow-up studies [24]. Thus, we proposed the use of zebrafish larvae as a simple and cost-effective in vivo model of VM. Although VM-like structures were observed in some xenografts, their formation should be interpreted with caution as there is no evidence indicating that they represent actual lumens. In addition, it is not clear why these structures were not formed in all xenografts. Such disparity in the formation of in vivo mimetic vessels has been nonetheless observed in murine xenografts [36]. However, several technical limitations may arise when using the larval zebrafish model. Firstly, there is a possibility of tumour cell leakage out of the fish due to poor resealing of the yolk sac membrane. Secondly, their smaller body size restricts the number of microinjected cells and the resulting tumour size compared with larger animal models. Finally, larval assays are performed at 34 °C, which may not be suitable for some cell lines and hence fail to form proper tumour colonies [38]. Further studies would be paramount to optimizing this model and testing its feasibility for real-time imaging as well as for therapeutic and functional assays.

## 5. Conclusions

In conclusion, our study provides a comprehensive comparative analysis of VM in HNSCC using a variety of experimental approaches. We, however, acknowledge some limitations, including the lack of perfusion assays to assess the functionality of the tubular networks, which has already been revealed in previous reports. Overall, our findings could offer a valuable resource for designing future studies that may facilitate the therapeutic exploitation of VM in HNSCC as well as in other recalcitrant tumours.

## Figures and Tables

**Table 1:** Vascular mimicry-like network formation for head and neck squamous cell carcinoma and human endothelial cell lines in two different matrices.

Matrigel				
**Cell density ^1^**	SCC-8	SCC-14	SCC-28	SCC-40
**A**	-	-	+	-
**B**	++	++	++	-
**C**	++	+++	+++	++
	SCC-44	SCC-73	SCC-81	SCC-106A
**A**	-	-	-	-
**B**	-	-	++	-
**C**	-	-	++	++
	SCC-25	SCC-24A	SCC-24B	HSC-3
**A**	-	+	+	+
**B**	-	+	++	++
**C**	-	+	+++	+++
	SAS		HUVEC	
**A**	+		+	
**B**	++		++	
**C**	+++		+++	
**Myogel**				
	SCC-8	SCC-14	SCC-28	SCC-40
**A**	+	-	+	-
**B**	+	+	++	-
**C**	+	+	+++	-
	SCC-44	SCC-73	SCC-81	SCC-106A
**A**	-	-	-	+
**B**	++	-	-	-
**C**	+++	-	-	-
	SCC-25	SCC-24A	SCC-24B	HSC-3
**A**	-	+	-	-
**B**	-	+	+	-
**C**	-	-	+	-
	SAS		HUVEC	
**A**	-		+	
**B**	-		++	
**C**	-		+++	

^1^ Data from one representative experiment of at least three independent experiments are shown; starting cell density was as follows: **A** = 20 × 10^3^; **B** = 40 × 10^3^; **C** = 60 × 10^3^ cells/well. Score description: (-) = no tube formation; (+) = poorly interconnected capillary networks that covered less than half of the matrix surface; (++) = cells formed well-defined interconnected capillary networks that covered not more than half of the matrix surface; (+++) = cells formed clear, well-defined interconnected capillary networks that covered more than half of the matrix surface. HUVEC: human umbilical vein endothelial cells.

## Data Availability

The datasets used in this study are available from the corresponding author upon a reasonable request.

## Supplementary Materials

The following are available online at https://www.mdpi.com/article/10.3390/cancers13194747/s1: Figure S1: Two primary cell lines (SCC-73 and SCC-25) were not able to initiate own tubulogenesis in either Matrigel or Myogel, where they remained dispersed as round cell aggregates (A, 40 × 10^3^ cells; B, 60 × 10^3^ cells); Table S1: Patient-derived cell lines and their corresponding data.
