# Supplementary material for: Comparative Analysis of Vascular Mimicry in Head and Neck Squamous Cell Carcinoma: In Vitro and In Vivo Approaches
Source: Cancers (Basel). 2021 Sep 23;13(19):4747. doi: 10.3390/cancers13194747 (PMC8507545; doi:10.3390/cancers13194747)
Supplement: Supplementary file 1 [file cancers-13-04747-s001.zip › cancers-1365128-supplementary.pdf]

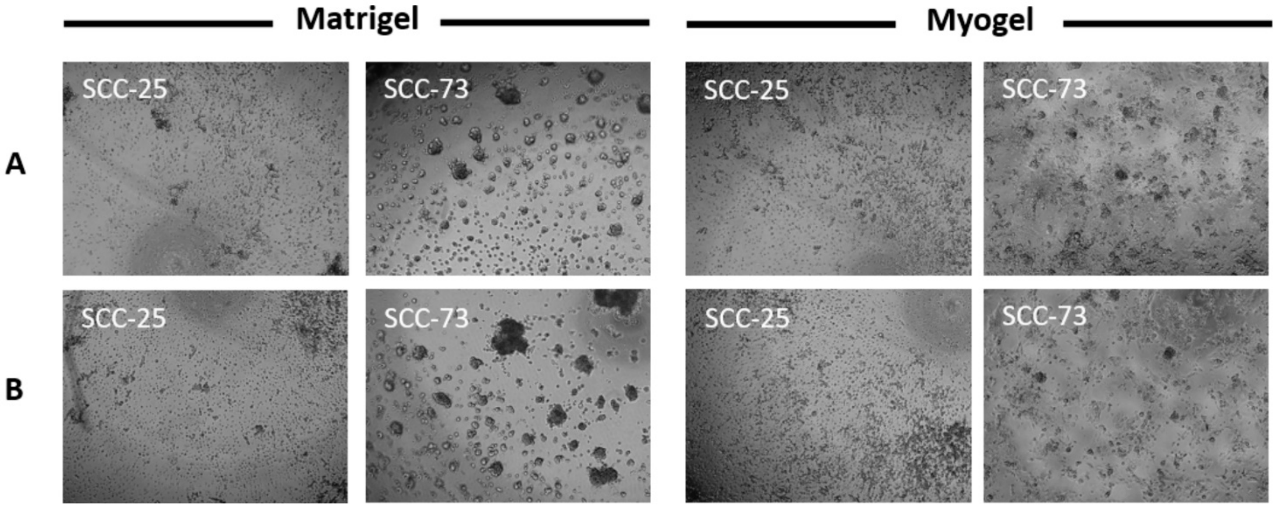

Figure S1. Two primary cell lines (SCC-73 and SCC-25) were not able to initiate own tubulogenesis in either Matrigel or Myogel, where they remained dispersed as round cell aggregates (A, 40 × 10^3 cells; B, 60 × 10^3 cells).

Table S1. Patient-derived cell lines and their corresponding data.

HNSCC cell line	Sex	Age (years)	TNM*	Site	Type	Grade	Passage**
**UT-SCC-8**	M	42	T2M0N0	Larynx	Pri	G1	51
**UT-SCC-14**	M	25	T3N1M0	Tongue	Pri	G2	33
**UT-SCC-24A**	M	41	T2N0M0	Tongue	Pri	G2	14
**UT-SCC-24B**	M	41	T2N1M0	Neck	Met	G2	15
**UT-SCC-28#**	F	48	T2N0M0	Floor of mouth	Pri	G1	30
**UT-SCC-40**	M	65	T3N0M0	Tongue	Pri	G1	10
**UT-SCC-44**	F	71	T4N2BM0	Gingiva	Pri	G3	33
**UT-SCC-73**	F	86	T1N0M0	Tongue	Pri	G2	16
**UT-SCC-81**	M	48	T2N0M0	Tongue	Pri	G1	16
**UT-SCC-106A**	M	37	T1N0M0	Larynx	Pri	G1	17

HNSCC, head and neck squamous cell carcinoma; M, male; F, female; Pri, primary tumour; Met, metastatic; TNM, tumour, lymph node, and distant metastasis staging. *TNM is based on the pathology report. **Indicates cell line passage number during the first experiment. # The primary tumour was treated with radiotherapy prior to the surgical resection.
